# Comprehensive evaluation of maize germplasm for alkali tolerance during germination

**DOI:** 10.3389/fpls.2025.1728607

**Published:** 2026-01-12

**Authors:** Zhuyun Pan, Zhiqiang Ge, Fengming Gao, Man Ao, Yixin Guan

**Affiliations:** 1 Northeast Institute of Geography and Agroecology, Chinese Academy of Sciences, Changchun, China; 2 College of Advanced Agricultural Sciences, University of Chinese Academy of Sciences, Beijing, China

**Keywords:** alkali tolerance, germplasm evaluation, LMG analysis, maize, seed germination

## Abstract

**Introduction:**

Alkaline stress poses a major challenge to crop productivity, often causing more severe physiological damage than saline stress alone. Maize is particularly sensitive to alkaline conditions, which significantly inhibit germination and early growth. The establishment of accurate evaluation systems for alkali tolerance is therefore crucial for the development of resilient cultivars.

**Methods:**

A total of 42 maize germplasm accessions were evaluated under simulated alkaline stress (100 mM) during germination. A comprehensive analytical framework integrating principal component analysis, membership function analysis, stepwise regression, cluster analysis, and discriminant analysis was used to assess alkalinity tolerance. The Lindeman–Merenda–Gold method was further employed to quantify the relative contribution of each morphological trait to the comprehensive alkali tolerance score.

**Results:**

Alkaline stress significantly inhibited early seedling growth, and several germination-related traits showed strong associations with alkali tolerance. Based on the comprehensive *D* value, the maize accessions were classified into five tolerance groups. Trait contribution analyses consistently indicated the germination index (GI) and the shoot dry weight (SDW) as the strongest determinants of tolerance, with root length (RL) and root fresh weight (RFW) also playing notable roles. These indicators form a reliable basis for the screening of alkali-tolerant maize germplasm, providing a foundation for future refinement of the evaluation system through physiological or molecular approaches.

**Discussion:**

This integrated evaluation system effectively distinguishes maize germplasm by alkali tolerance level and identifies key morphological determinants. The findings provide a scientific basis for germplasm screening and breeding of alkali-tolerant maize materials, contributing to sustainable agricultural production in saline–alkaline environments.

## 1 Introduction

Saline–alkali land is a valuable reserve of arable land resources, and its sustainable utilization has become a major research focus in recent years (Baloch et al., 2023). Among these, soda saline–alkali soils are widely recognized as the most challenging type to remediate, which are typically characterized by weak structural stability, high bulk density, surface crusting, and poor permeability, all of which severely inhibit plant growth and development (Nelson et al., 1998). Furthermore, climate change and human activities have exacerbated biodiversity loss and reduced productivity, making the management and restoration of saline–alkali land increasingly challenging (Sahab et al., 2021; Sarath et al., 2021). Against this backdrop, planting salt- and alkali-tolerant species, particularly those adapted to alkaline soils, has been demonstrated to effectively enhance soil productivity, reduce salinity, and improve soil structure (Liang and Shi, 2021). This approach serves as not only a crucial strategy to mitigate the arable land crisis (Rezvi et al., 2022) but also a key priority in saline–alkali land research.

As a major global food and feed crop, maize accounts for approximately one-third of the total global grain production (Ali et al., 2022; McMillen et al., 2022) and is relatively vulnerable to saline–alkali stress. Plant stress tolerance assessments typically focus on the stress responses across developmental stages, with the germination and seedling stages being particularly sensitive to environmental stresses and, thus, critical for the early-stage screening of stress tolerance (Dodd and Donovan, 1999). Research on stress tolerance during these stages not only provides theoretical support for vegetation restoration in saline–alkali soils but also offers crucial evidence for the early identification of stress-tolerant materials in breeding programs (Ren et al., 2025; Hu et al., 2025; Zheng et al., 2023). Therefore, systematically evaluating the salt tolerance of maize seedlings and identifying the key tolerance traits hold significant practical importance for the development of more adaptable salt-tolerant maize germplasm resources.

**Figure 1:**
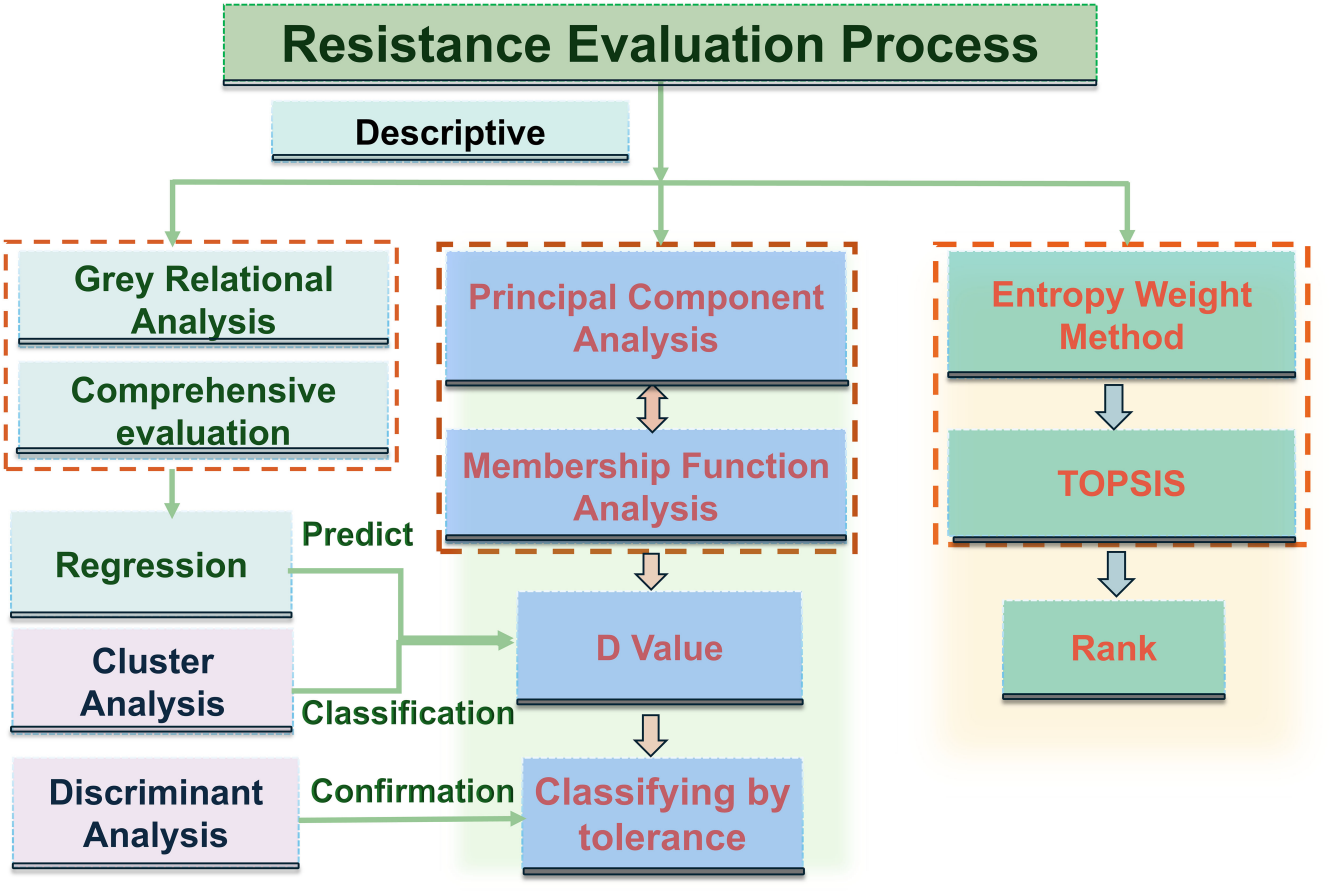
Tolerance evaluation flowchart. Colored background indicates the selection process for tolerant materials. The methods shown in the figure are not limited to those listed. Other analytical methods that perform the same function can be used instead.

Robust tolerance evaluation further requires statistical tools that can identify critical traits and reliably classify germplasm. Multivariate analytical methods—including principal component analysis (PCA), cluster analysis (CA), entropy weighting, fuzzy evaluation, grey relational analysis, and TOPSIS (technique for order of preference by similarity to ideal solution)—have been widely applied in crops such as rice (Zhang et al., 2025), wheat (Yang et al., 2025), soybean (Zhao et al., 2022), and potato (Cao et al., 2015) and have also been proven useful in diverse research fields, including food science (Hosseinpour and Martynenko, 2021), ecology (Gonzalez-Martinez et al., 2021), and medicine (Królaczyk et al., 2020), highlighting their versatility in analyzing complex biological systems (**Figure 1**). However, despite their broad use, these approaches often offer limited interpretability of individual variables and lack explicit validation of their classification performance or quantitative partitioning of trait contributions (Han et al., 2019; Yu et al., 2021; Zhang et al., 2022; Zhao et al., 2023; Song et al., 2024; Baha et al., 2025). Linear discriminant analysis (LDA) can address these gaps by verifying the clustering accuracy (Helm and Eis, 2007; Caturegli et al., 2020; Tang et al., 2024), while the Lindeman–Merenda–Gold (LMG) method decomposes the model *R*^2^ to determine the relative importance of each trait (Lindeman et al., 1980). These tools have shown strong utility in plant ecology, grassland productivity assessment, and crop stress physiology (Ding et al., 2025; Liu et al., 2024; Wu et al., 2024; Martini et al., 2019; Kamphorst et al., 2021), providing a robust and interpretable framework for the evaluation of stress tolerance across biological systems.

Despite these methodological advances, applications in maize remain limited. The majority of the evaluations of alkalinity tolerance employed single or partially integrated analytical strategies, and the contributions of individual early-stage morphological traits to tolerance variations have not been quantitatively resolved. A unified analytical framework that integrates multivariate characterization, classification validation, and trait importance assessment for the alkalinity responses during the germination stage is still lacking.

Building on germplasm studies in rice, wheat, soybean, potato, and bermudagrass, which have demonstrated the analytical value of the integration of multiple statistical perspectives, we extend these approaches to maize alkalinity tolerance. We hypothesize that early morphological traits differ in their contributions to tolerance differentiation, an aspect that has not yet been resolved quantitatively. To address this, we quantified the germination responses under alkaline stress, identified the most informative morphological indicators, and developed an integrated and explicitly data-driven evaluation framework. By combining PCA, membership function analysis (MFA), CA, stepwise regression, LDA, LMG-based trait importance assessment, and *t*-tests into a coherent analytical pipeline, this study introduces a novel, multi-tiered system that unifies characterization, classification, and trait importance inference. This integrative strategy advances the current screening methodologies and provides an innovative platform for accelerating the development of maize lines with improved alkalinity tolerance.

## 2 Materials and methods

### 2.1 Experimental materials

A total of 42 maize accessions were evaluated in this study, all derived from segregating populations of the cross between ZD06, a salt- and alkali-sensitive variety, and NM193, a highly salt- and alkali-tolerant variety. The specific tolerance of each accession was not known prior to the experiment and was assessed through germination and early seedling evaluations under alkaline stress. All accessions, except Xianyu335, were provided by the Maize Genetics and Molecular Breeding Laboratory at the Northeast Institute of Geography and Agroecology. Xianyu335 was purchased online. Full details of all 42 accessions, including the genetic type and parental lines, are provided in **Table 1**.

**Table 1:** Materials tested.

Name	Maize accession	Parental line	Genetic type	Name	Maize accession	Parental line	Genetic type
ZM1	YTEST10601055	ZD06×NM193	Segregating population	ZM22	YTEST10601041	ZD06×NM193	Segregating population
ZM2	YTEST10591058	ZD06×NM193	Segregating population	ZM23	YTEST0591052	ZD06×NM193	Segregating population
ZM3	YTEST10591064	ZD06×NM193	Segregating population	ZM24	YTEST0591062	ZD06×NM193	Segregating population
ZM4	YTEST10601034	ZD06×NM193	Segregating population	ZM25	YTEST0601046	ZD06×NM193	Segregating population
ZM5	YTEST10601024	ZD06×NM193	Segregating population	ZM26	YTEST0601058	ZD06×NM193	Segregating population
ZM6	YTEST10591057	ZD06×NM193	Segregating population	ZM27	YTEST0601033	ZD06×NM193	Segregating population
ZM7	YTEST0601026	ZD06×NM193	Segregating population	ZM28	YTEST0601037	ZD06×NM193	Segregating population
ZM8	YTEST0601011	ZD06×NM193	Segregating population	ZM29	YTEST0601062	ZD06×NM193	Segregating population
ZM9	YTEST0601049	ZD06×NM193	Segregating population	ZM30	YTEST10561045	ZD06×NM193	Segregating population
ZM10	YTEST0601020	ZD06×NM193	Segregating population	ZM31	YTEST10561042	ZD06×NM193	Segregating population
ZM11	YTEST0601016	ZD06×NM193	Segregating population	ZM32	Xianyu335	–	–
ZM12	YTEST0601013	ZD06×NM193	Segregating population	ZM33	YTEST0621028	ZD06×NM193	Segregating population
ZM13	YTEST10601012	ZD06×NM193	Segregating population	ZM34	YTEST0621029	ZD06×NM193	Segregating population
ZM14	YTEST10601006	ZD06×NM193	Segregating population	ZM35	YTEST0621030	ZD06×NM193	Segregating population
ZM15	YTEST10601004	ZD06×NM193	Segregating population	ZM36	YTEST0621020	ZD06×NM193	Segregating population
ZM16	YTEST10591055	ZD06×NM193	Segregating population	ZM37	YTEST0621021	ZD06×NM193	Segregating population
ZM17	YTEST10601009	ZD06×NM193	Segregating population	ZM38	YTEST10551057	ZD06×NM193	Segregating population
ZM18	YTEST10591053	ZD06×NM193	Segregating population	ZM39	YTEST10551064	ZD06×NM193	Segregating population
ZM19	YTEST10601025	ZD06×NM193	Segregating population	ZM40	YTEST10561058	ZD06×NM193	Segregating population
ZM20	YTEST10601007	ZD06×NM193	Segregating population	ZM41	YTEST10601050	ZD06×NM193	Segregating population
ZM21	YTEST10591060	ZD06×NM193	Segregating population	ZM42	YTEST10561047	ZD06×NM193	Segregating population

### 2.2 Experimental design

#### 2.2.1 Germination assay under alkaline stress

A completely randomized design (CRD) was used to assess the maize germination performance under alkaline stress. Two treatments were established: 1) control, which used distilled water, and 2) alkaline stress, which used a mixed alkaline solution of Na_2_CO_3_/NaHCO_3_ = 1:9 at 100 mM. Each treatment contained three replicates, with 10 seeds per replicate, resulting in 60 experimental units in total (2 treatments × 3 replicates × 10 seeds). Uniform and fully developed seeds were carefully selected for the experiment. The seeds were surface-sterilized by immersion in anhydrous ethanol for 60 s, followed by three to five rinses with tap water and distilled water. The seeds were then immersed in 1% NaClO for 15 min, rinsed thoroughly with distilled water, air-dried, and stored for subsequent use. For the germination assays, the seeds were placed in 9-cm Petri dishes lined with a double-layered filter paper, with each dish corresponding to one experimental unit. The dishes were incubated in a controlled growth chamber under a 14-h/10-h light/dark cycle, with temperatures maintained at 25 °C/20 °C (day/night). Germination was recorded when the radicle protruded ≥2 mm, starting on day 3 and continuing for 7 days. Daily weighing was conducted to maintain constant solution volume, and evaporated water was replenished with the corresponding treatment solution.

#### 2.2.2 Measurement and statistics of the morphological index

After 7 days of incubation, 11 germination and early seedling traits were measured for each treatment: the following formulas were employed to ascertain the germination potential (GP), the germination rate (GR), the germination index (GI), and the vigor index (VI). The amount of seedling growth [root length (RL) + shoot length (SL)] was multiplied by the GI to obtain the VI (Zhou et al., 2023). Calipers were utilized to measure the RL and SL, the samples were weighed on a balance, dried in an oven at 105 °C for 6 h, and then reweighed. The alkali tolerance coefficient (ATC) was determined for each indication (refer to Equation 6).

##### 2.2.2.1 Germination potential

(1) $$GP=\frac{number\;of\;seeds\;ger\min ated\;on\;day\;3}{number\;of\;seeds\;tested}$$

GP refers to the proportion of seeds that have successfully germinated by the third day relative to the total number of seeds. The germination energy of the tested materials was calculated using Equation 1.

##### 2.2.2.2 Germination rate

(2) $$GR=\frac{number\;of\;seeds\;ger\min ated\;on\;day\;7}{number\;of\;seeds\;tested}$$

GR refers to the proportion of seeds that have successfully germinated by the seventh day relative to the total number of seeds. The GR of the tested materials was calculated using Equation 2.

##### 2.2.2.3 Germination index

(3) $$GI=\sum \;\frac{Gt}{Dt}$$

The GI of the tested materials was calculated using Equation 3, where *G_t_* refers to the number of seeds germinated within *t* days and *D_t_* represents the corresponding germination days.

##### 2.2.2.4 Vigor index

(4) VI=GI·S

The VI of the tested materials was calculated using Equation 4, where GI represents the germination index and *S* represents seedling growth.

##### 2.2.2.5 Root-to-shoot ratio

(5) $$RSR=\frac{Root\;fresh\;weight}{Shoot\;fresh\;weight}$$

The root-to-shoot ratio (RSR) of the tested materials was calculated using Equation 5.

### 2.3 Data analysis

#### 2.3.1 Analysis

In this study, we integrated correlation analysis, PCA, MFA, stepwise regression, CA, LDA, and LMG-based variable importance analysis to construct a multidimensional and systematic evaluation framework, thereby enabling a more comprehensive, robust, and accurate screening of germplasm resources.

##### 2.3.1.1 Alkali tolerance indicator for various indicators of maize

According to Yu et al. (2021), and with suitable amendments, analysis was performed using the relative values of each maize material, and based on this, the ATC for each indicator was calculated.

(6) $$ATC=\frac{X_{ij-treat}}{X_{ij-control}}$$

where ATC*_ij_* is the alkali tolerance coefficient of the indicator (*j*) for variety (*i*). *X_ij_*_-control_ and *X_ij_*_-treat_ represent the indicator values of each variety assessed under the no-alkali treatment and the alkali treatment, respectively.

##### 2.3.1.2 Membership function values for the comprehensive indicators of maize

(7) $$\mu \left(X_{i}\right)=\frac{X_{i}-X_{i\min}}{X_{i\max}-X_{i\min}}$$

The membership function value *μ* was used to evaluate the alkali tolerance of multiple indicators, which was calculated using Equation 7.

##### 2.3.1.3 Weights of the comprehensive indicators

(8) $$W_{i}=\frac{P_{i}}{\sum_{i=1}^{n}P_{i}\;i=1,2,3\ldots \ldots n}$$

**Equation 8** was used to calculate the weight function *W_i_*, which represents the relative importance of the comprehensive indicators for a variety. *P_i_* denotes the contribution of the comprehensive indicators.

##### 2.3.1.4 Alkali tolerance of maize germplasm resources

(9) $$D=\sum_{i=1}^{n}\left[\mu (X_{i})\times W_{i}\right]\;\;\;i=1,2,3\ldots \ldots n$$

The comprehensive evaluation parameter (*D*) for the alkali tolerance resilience of each variety was calculated using Equation 9 to assess the alkali tolerance of the different maize materials.

##### 2.3.1.5 Relative contribution

Standardized regression coefficients (*β*) were used to represent the relative effect size of each variable on the dependent variable. The contribution rate of each indicator was calculated as follows:

(10) $$C_{i}=\frac{\left|\beta_{i}\right|}{\sum_{j}^{n}|\beta_{i}|}$$

where *C_i_* represents the relative contribution (in percent) of the *i*-th indicator and *β_i_* is the standardized regression coefficient obtained from the regression model. This method provides an intuitive estimation of the relative importance of predictors and has been widely applied in quantitative studies evaluating complex traits (Nathans et al., 2012).

##### 2.3.1.6 LMG method

To quantify the relative contribution of each indicator to maize alkali tolerance, this study employed the LMG method.

(11) $$Y=\beta_{0}+\sum_{i=1}^{p}\beta_{i}X_{i}+\epsilon$$

where *Y* is the response variable, *X_i_* is the explanatory variable, *β*_a_ is the regression coefficient, and *ϵ* is the residual. LMG calculates the average contribution of each variable to the total *R*^2^ (LMG_i_) by considering all possible variable entry orders. The relative contribution of each indicator to the model was assessed using the LMG metric implemented in the relaimpo package in R (Groemping, 2006).

Data are presented as the mean ± SE. Data reduction was performed using Excel 24. PCA, regression analysis, correlation analysis, CA, and discriminant analysis were performed using IBM SPSS Statistics version 26. The relative contributions of the variables (LMG) were calculated in R 4.4.3 using the relaimpo package [calc.relimp(), type = “lmg”, rela = TRUE]. Hierarchical clustering with significance annotation was conducted via the online platform OmicStudio (https://www.bioinformatics.com.cn, last accessed August 18, 2025). All other figures were generated using Origin 25.

## 3 Results

### 3.1 Genetic analysis of the alkali tolerance coefficients for morphological traits in 42 maize materials

Extensive genetic variation is a fundamental prerequisite for the effective screening of alkaline-tolerant maize materials (**Table 2**). Alkaline stress exerts a pronounced effect on the germination traits of maize, inducing variations across 11 measured parameters, with coefficients of variation ranging from 45% (RSR) to 95% (GP). These results indicate substantial diversity in the responses of maize genotypes to alkaline stress, thereby providing a robust basis for the identification and selection of salt- and alkali-tolerant germplasm.

**Table 2:** Summary statistics for maize germination stages.

Trait	Maximum	Minimum	Range	Standard deviation	Coefficient of variation
GP	1.83	0.00	1.83	0.43	0.95
GR	1.31	0.15	1.16	0.29	0.52
GI	1.78	0.11	1.68	0.37	0.63
VI	0.67	0.01	0.66	0.17	0.83
RSR	1.87	0.31	1.57	0.31	0.45
RL	0.76	0.04	0.72	0.15	0.70
SL	1.43	0.14	1.29	0.31	0.56
RFW	1.62	0.05	1.57	0.34	0.78
SFW	1.92	0.11	1.81	0.40	0.64
RDW	1.99	0.03	1.96	0.39	0.78
SDW	1.97	0.10	1.87	0.37	0.60

*GP*, germination potential; *GR*, germination rate; *GI*, germination index; *VI*, vigor index; *RSR*, root-to-shoot ratio; *RL*, root length; *SL*, shoot length; *RFW*, root fresh weight; *SFW*, shoot fresh weight; *RDW*, root dry weight; *SDW*, shoot dry weight.

### 3.2 Coefficients of the morphological traits in maize during germination under alkaline stress

**Figure 2:**
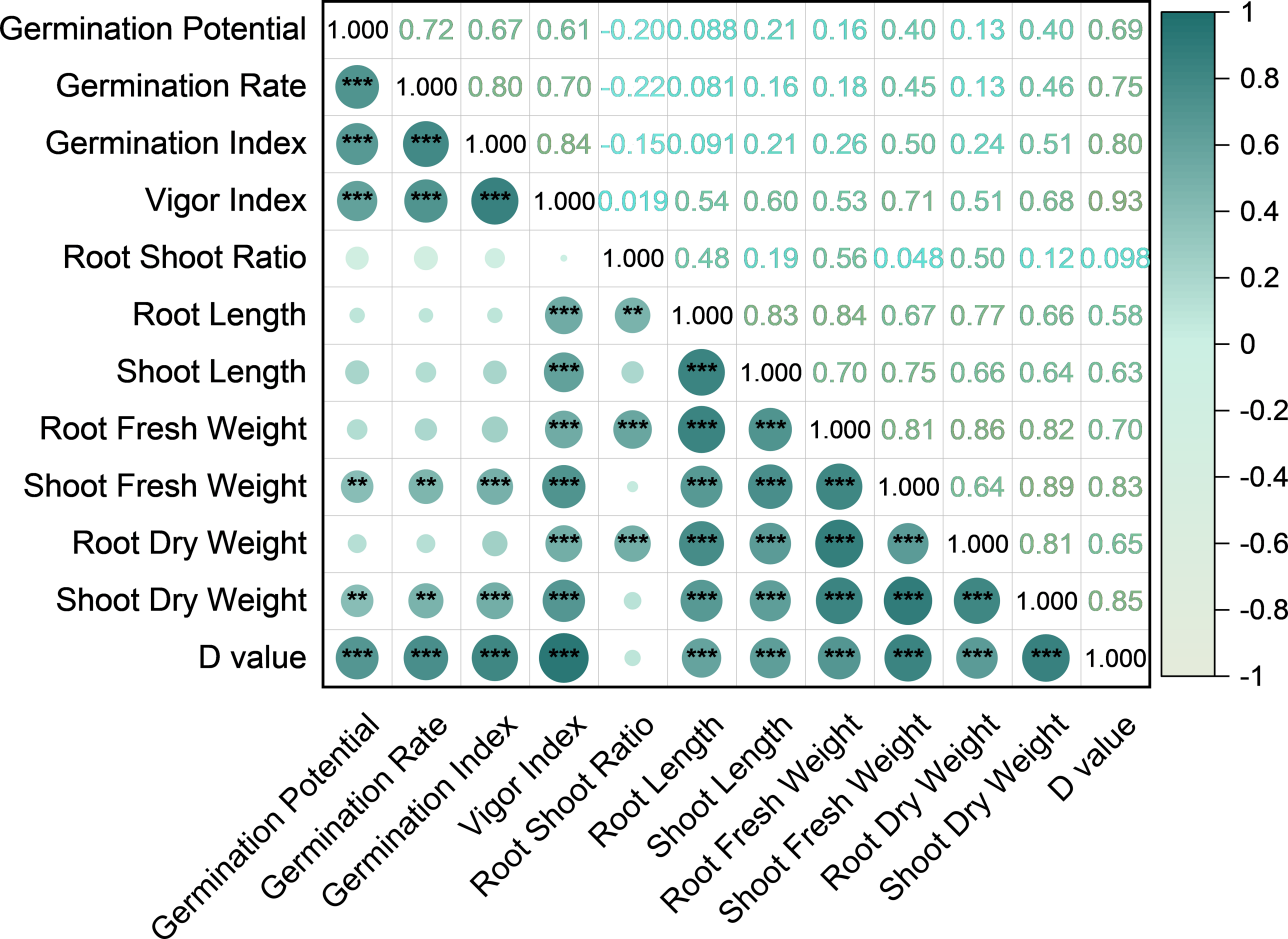
Correlation of the salt tolerance coefficients among traits, correlation of the salt tolerance coefficient of each trait, and comprehensive evaluation of the *D* value. *p < 0.05, **p < 0.01, ***p < 0.001.

A correlation analysis was conducted on the ATCs of all the measured indicators. The results are presented in **Figure 2**. GP showed highly significant positive correlations (*p* < 0.01) with GR, GI, VI, shoot fresh weight (SFW), and shoot dry weight (SDW), indicating that a higher GP reflects an enhanced early germination and seedling vigor under alkaline stress. In contrast, GP was not significantly correlated (*p* > 0.05) with the RSR, RL, SL, root fresh weight (RFW), or root dry weight (RDW), suggesting that GP primarily reflects aboveground early growth rather than root development. GR and GI showed similar correlation patterns to GP, emphasizing their roles in capturing early germination dynamics. VI was significantly correlated (*p* < 0.01) with the majority of the traits, reflecting its integrative representation of the overall seedling vigor. RSR correlated strongly with RL, RFW, and RDW, highlighting its function in root allocation. RL, along with RFW, RDW, SL, and SDW, showed strong intercorrelations, indicating coordinated development of the root and shoot biomass, which is critical for seedling establishment under alkaline stress. These results demonstrate that the 11 morphological indicators (i.e., GP, GR, GI, VI, RSR, RL, SL, RFW, SFW, RDW, and SDW) capture distinct aspects of the maize seedling responses to alkaline stress, including early germination, root–shoot allocation, and biomass accumulation, providing a detailed understanding of the physiological and morphological effects of alkalinity.

### 3.3 Principal component analysis and membership function analysis of the alkali tolerance indicators in different maize materials

PCA was conducted to reduce the dimensionality of the ATCs under the different salt stress concentrations (**Table 3**). Factor analysis of the 11 original indicators yielded three principal components (PC1, PC2, and PC3), which accounted for 54.4%, 24.35%, and 7.45% of the total variance, respectively. Collectively, these three components explained 86.2% of the overall variance, effectively capturing the majority of information contained in the original indicators. Subsequently, membership functions were applied to calculate composite scores for the three principal components, which were used for a comprehensive evaluation of alkali tolerance (**Table 4**). The membership function (*μ*) values for the composite score of each variety were calculated according to Equation 7. As shown in **Table 4**, these *μ*(*X_i_*) values reflect the relative alkali tolerance of each variety, where *μ*(*X_i_*) = 1 represents the highest tolerance and *μ*(*X_i_*) = 0 indicates the highest sensitivity. For the Pu1 composite component, ZM42 exhibited the strongest alkali tolerance, whereas ZM17 was the most sensitive. For Pu2, ZM40 showed the highest tolerance, while ZM42 was the most sensitive. For Pu3, ZM14 demonstrated the greatest tolerance, while ZM6 was the most sensitive. Because the rankings based on the three composite indicators occasionally differed, an integrated assessment was performed to provide a more reliable basis for the selection of alkali-tolerant maize materials.

**Table 3:** Eigenvalues representing the contribution of maize traits extracted using principal component analysis.

Trait	PC1	PC2	PC3
GP	0.20	0.41	0.15
GR	0.22	0.45	0.20
GI	0.25	0.42	0.25
VI	0.35	0.23	0.07
RSR	0.11	−0.41	0.75
RL	0.32	−0.30	−0.10
SL	0.32	−0.16	−0.40
RFW	0.35	−0.25	0.11
SFW	0.37	0.01	−0.31
RDW	0.33	−0.25	0.13
SDW	0.37	−0.01	−0.13
Eigenvalue	5.98	2.68	0.82
Contribution (%)	54.40	24.35	7.45
Cumulative contribution (%)	54.40	78.75	86.20
Weight coefficient (%)	63.10	28.25	8.64

*GP*, germination potential; *GR*, germination rate; *GI*, germination index; *VI*, vigor index; *RSR*, root-to-shoot ratio; *RL*, root length; *SL*, shoot length; *RFW*, root fresh weight; *SFW*, shoot fresh weight; *RDW*, root dry weight; *SDW*, shoot dry weight.

**Table 4:** Membership function values of maize.

Species	Pu(X1)	Pu(X2)	Pu(X3)	Species	Pu(X1)	Pu(X2)	Pu(X3)
ZM1	0.43	0.48	0.08	ZM22	0.16	0.60	0.54
ZM2	0.72	0.83	0.46	ZM23	0.21	0.54	0.44
ZM3	0.25	0.64	0.34	ZM24	0.37	0.77	0.35
ZM4	0.56	0.71	0.22	ZM25	0.12	0.59	0.36
ZM5	0.78	0.75	0.55	ZM26	0.47	0.79	0.45
ZM6	0.50	0.38	0.00	ZM27	0.53	0.39	0.61
ZM7	0.48	0.49	0.32	ZM28	0.45	0.62	0.63
ZM8	0.15	0.74	0.42	ZM29	0.55	0.66	0.54
ZM9	0.04	0.60	0.44	ZM30	0.40	0.38	0.72
ZM10	0.95	0.64	0.53	ZM31	0.34	0.50	0.22
ZM11	0.35	0.61	0.59	ZM32	0.44	0.36	0.68
ZM12	0.27	0.56	0.38	ZM33	0.79	0.36	0.21
ZM13	0.06	0.56	0.38	ZM34	0.29	0.45	0.63
ZM14	0.39	0.16	1.00	ZM35	0.48	0.46	0.62
ZM15	0.04	0.61	0.40	ZM36	0.25	0.44	0.50
ZM16	0.18	0.60	0.63	ZM37	0.29	0.65	0.54
ZM17	0.00	0.51	0.39	ZM38	0.58	0.82	0.49
ZM18	0.30	0.53	0.66	ZM39	0.77	0.94	0.59
ZM19	0.05	0.51	0.60	ZM40	0.63	1.00	0.73
ZM20	0.20	0.67	0.52	ZM41	0.53	0.52	0.40
ZM21	0.10	0.57	0.40	ZM42	1.00	0.00	0.38

### 3.4 Comprehensive evaluation of maize alkali tolerance

The comprehensive trait (*μ* value) was calculated using the membership function method, and the *D* value was determined based on the weights obtained from the PCA (Equations 8, 9). A higher *D* value indicates stronger alkali tolerance, whereas a lower *D* value reflects weaker tolerance. The comprehensive scores and the rankings of the germination traits for 42 maize materials were thus determined. Higher comprehensive scores corresponded to greater tolerance to saline–alkaline stress, and all materials were ranked from the highest to the lowest accordingly (**Table 5**). The *D* values ranged from 0.82 to 0.18. Material ZM10 ranked first, with a *D* value of 0.82, indicating superior performance in the alkali tolerance evaluation system. In contrast, material ZM17 had the lowest *D* value (0.18), ranking last and exhibiting high sensitivity to alkaline stress.

**Table 5:** Comprehensive assessment of alkali tolerance using the D value and the predicted D′ value.

Species	*D* value	$D^{\prime }$value	Ranking	Species	*D* value	$D^{\prime }$ value	Ranking
ZM10	0.82	0.85	1	ZM1	0.41	0.41	22
ZM39	0.80	0.79	2	ZM37	0.41	0.44	23
ZM5	0.75	0.74	3	ZM18	0.40	0.41	24
ZM40	0.74	0.75	4	ZM31	0.38	0.40	25
ZM2	0.73	0.72	5	ZM14	0.38	0.38	26
ZM42	0.66	0.68	6	ZM3	0.37	0.39	27
ZM38	0.64	0.64	7	ZM34	0.36	0.35	28
ZM33	0.62	0.61	8	ZM20	0.36	0.37	29
ZM29	0.58	0.57	9	ZM12	0.36	0.36	30
ZM4	0.57	0.56	10	ZM8	0.34	0.35	31
ZM26	0.56	0.56	11	ZM16	0.34	0.33	32
ZM41	0.51	0.48	12	ZM36	0.32	0.30	33
ZM28	0.51	0.53	13	ZM23	0.32	0.33	34
ZM27	0.50	0.50	14	ZM22	0.32	0.32	35
ZM35	0.48	0.48	15	ZM25	0.27	0.27	36
ZM24	0.48	0.49	16	ZM21	0.26	0.25	37
ZM7	0.47	0.45	17	ZM9	0.23	0.23	38
ZM11	0.45	0.46	18	ZM15	0.23	0.24	39
ZM32	0.44	0.43	19	ZM19	0.23	0.23	40
ZM6	0.42	0.40	20	ZM13	0.23	0.24	41
ZM30	0.42	0.43	21	ZM17	0.18	0.17	42

Species indicates maize germplasm identifiers. D value represents the comprehensive evaluation score of alkali tolerance, and D′ value represents the predicted comprehensive score.

### 3.5 Screening key indicators for alkalinity tolerance using stepwise regression

Stepwise regression analysis was conducted to examine the relationship between several indicators and the ATC of maize. The candidate variables identified through the initial correlation analysis were used in the stepwise regression, with the *D* value as the dependent variable and the substantially associated indices as the independent variables for each variety. The resulting optimal regression equation is as follows:

(12) $$D^{\prime }=0.109+0.091SDW+0.169GI+0.073GP+0.26RL+0.085RFW+0.102GR$$

**Figure 3:**
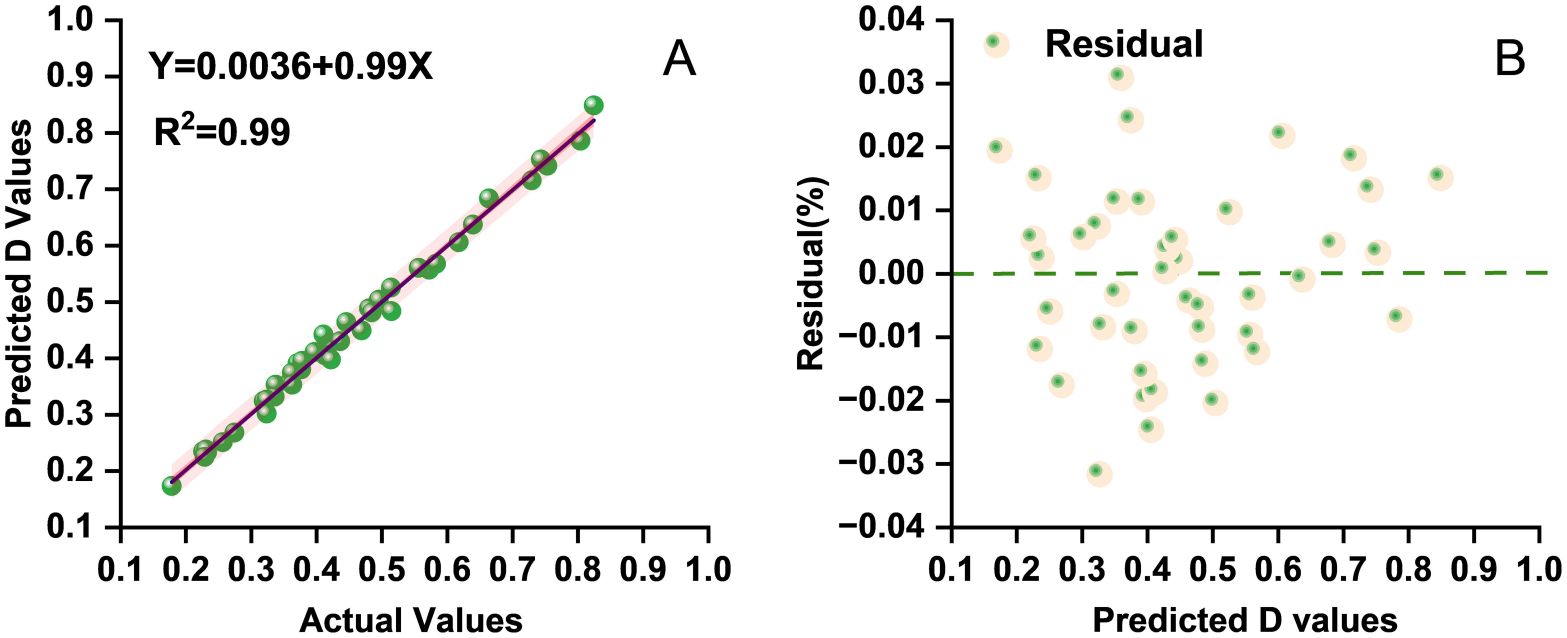
**(A)** Scatter distribution diagram of the predicted and the actual values on the test set for multiple stepwise regression. **(B)** Residual scatter plot from the stepwise regression analysis showing the distribution of residuals for the assessment of model fit and variance homogeneity.

Seven indicators (i.e., GI, SDW, RFW, GR, GP, and RL) were incorporated due to their strong linear correlations with the *D* value. Model performance was evaluated through residual analysis and comparison of the predicted *versus* the observed values (**Figure 3**). The close alignment of the predicted and the observed values along the diagonal line indicates high predictive accuracy. The regression analysis results (**Tables 5**, **6**) further confirmed a good overall model fit and statistical significance (*F* = 810.617, ***p < 0.001). These findings demonstrate that the stepwise regression model can reliably predict the alkali tolerance of maize based on the *D* value and that the selected morphological traits can serve as effective indicators for the evaluation and screening of maize germplasm.

**Table 6:** Results of the stepwise linear regression analysis.

Variable	Non-standardized coefficients		Standardized coefficients			Collinearity statistics	
	*B*	Standardized error	Beta	*t*	Sig.	Tolerance	VIF
Constant	0.109	0.006		17.429	0.000***	0.214	4.678
SDW	0.091	0.014	0.203	6.559	0.000***	0.309	3.235
GI	0.169	0.012	0.371	14.422	0.000***	0.28	3.566
RL	0.260	0.029	0.240	8.901	0.000***	0.455	2.196
GP	0.073	0.008	0.188	8.868	0.000***	0.289	3.466
GR	0.102	0.015	0.176	6.615	0.000***	0.162	6.158
RFW	0.085	0.017	0.173	4.878	0.000***	0.214	4.678
*R* ^2^	0.993						
Adj.*R*^2^	0.992						
*F*	*F* = 810.617, ***p = 0.000						

*SDW*, shoot dry weight; *GI*, germination index; *RL*, root length; *GP*, germination potential; *GR*, germination rate; *RFW*, root fresh weight.

*p < 0.05, **p < 0.01, ***p < 0.001.

### 3.6 Relative importance analysis based on the LMG method

**Figure 4:**
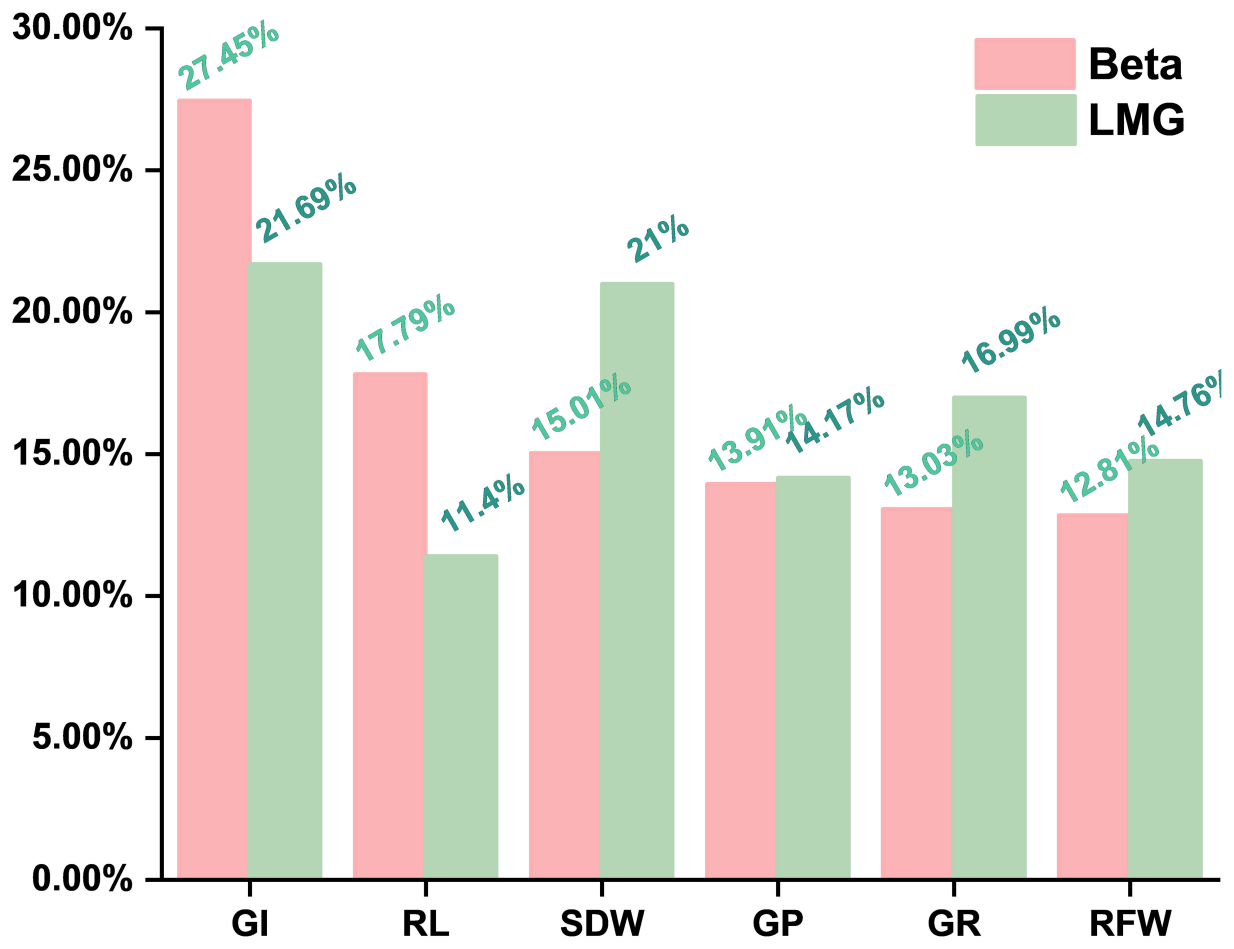
Standardized beta and Lindeman–Merenda–Gold (LMG) contribution, The relative influence of the key traits on alkali tolerance was quantified using standardized beta coefficients and LMG contributions, providing a reliable basis for the screening of alkali-tolerant maize germplasm.

Stepwise regression identified six key traits (i.e., SDW, GI, RL, GP, GR, and RFW) for the construction of the maize alkalinity tolerance model. The standardized beta coefficients indicated (Equation 10) that GI had the strongest direct effect on *D* (0.37, 27.45%), followed by RL (0.24, 17.79%) and SDW (0.21, 15.01%), while GP, GR, and RFW had smaller direct effects (**Table 7**). LMG analysis (Equation 11) revealed that GI, SDW, and GR contributed most to the model’s explanatory power (21.69%, 21.00%, and 16.99%), whereas RFW, GP, and RL contributed 14.76%, 14.17%, and 11.40%, respectively (**Table 7**, **Figure 4**). The combined contribution of the root traits (RFW + RL, 26.16%) underscores the substantial role of root growth in determining alkalinity tolerance. Moreover, the germination-related traits, particularly GI, along with SDW, showed both strong direct effects and significant overall contributions, highlighting the importance of early seedling vigor and biomass accumulation. The agreement between the beta and LMG results indicates that GI and SDW play dominant roles, whereas the root traits and the other germination indicators provide supportive contributions. These findings provide a quantitative basis for the evaluation of maize seedlings and suggest that GI, SDW, and the root traits should be prioritized when screening germplasm and when selecting materials with enhanced alkalinity tolerance, offering a robust framework to guide breeding programs and improve selection efficiency.

**Table 7:** Standardized beta and Lindeman–Merenda–Gold (LMG) contribution.

Variable	Beta	Beta contribution (%)	LMG contribution (%)
GI	0.37	27.45	21.69
RL	0.24	17.79	11.40
SDW	0.20	15.01	21.00
GP	0.19	13.91	14.17
GR	0.18	13.03	16.99
RFW	0.17	12.81	14.76

*GI*, germination index; *RL*, root length; *SDW*, shoot dry weight; *GP*, germination potential; *GR*, germination rate; *RFW*, root fresh weight.

### 3.7 Cluster analysis of maize materials

**Figure 5:**
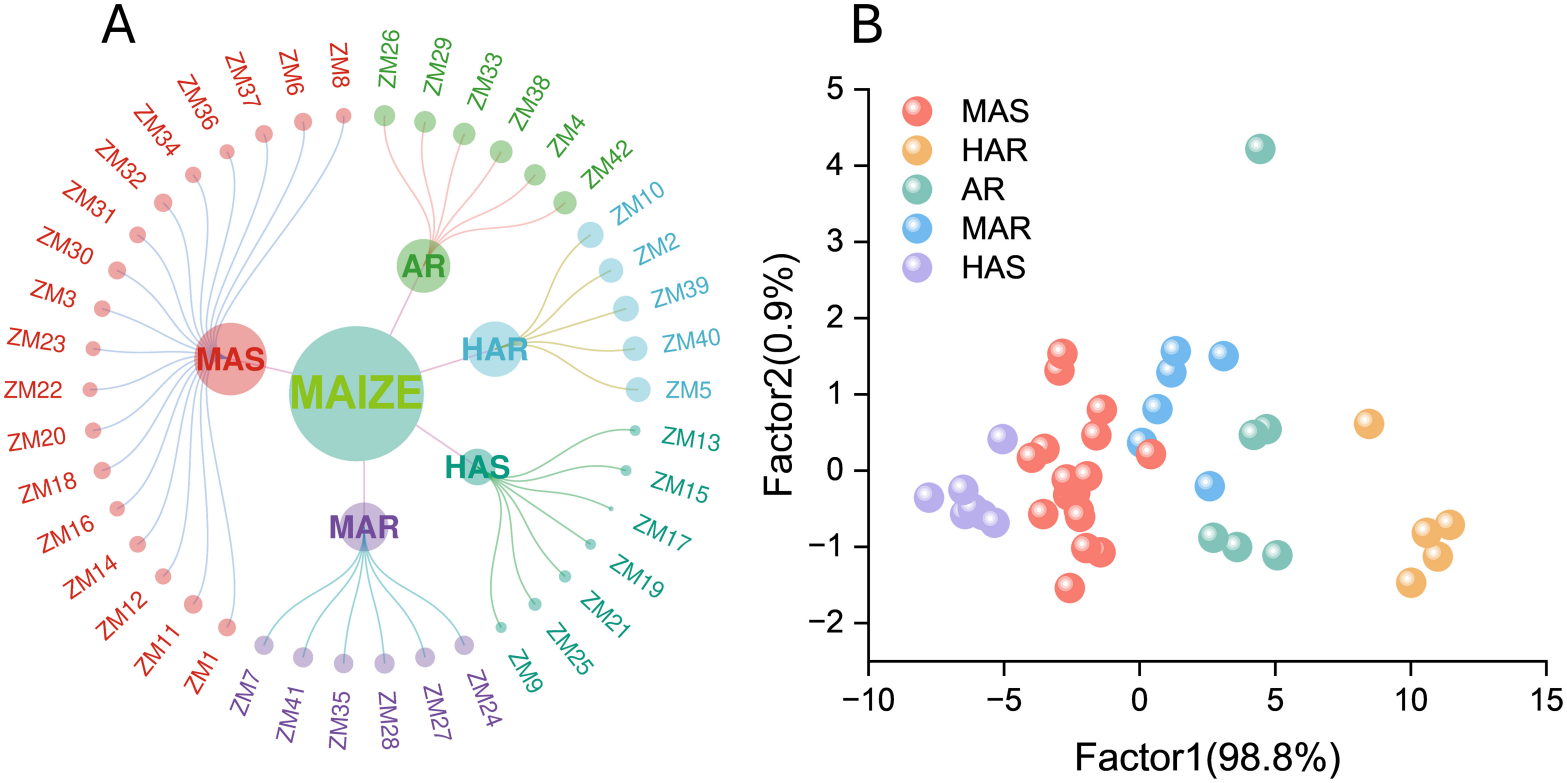
**(A)** The 42 maize materials classified into five groups via cluster analysis: high alkali tolerance, alkali tolerance, general alkali tolerance, general alkali sensitivity, and high alkali sensitivity. **(B)** Scatter plot of the discriminant analysis for the maize germplasm categories showing the separation of different maize lines based on the measured traits.

Systematic clustering was conducted using intergroup connectivity and squared Euclidean distance, with the composite *D* value as the primary clustering criterion. The 42 maize accessions were classified into five distinct groups (**Figure 5A**): highly alkali-resistant (HAR, five accessions), alkali-resistant (AR, six accessions), moderately alkali-resistant (MAR, six accessions), moderately alkali-sensitive (MAS, 18 accessions), and highly alkali-sensitive (HAS, seven accessions). MAS represented the largest proportion. To further illustrate the phenotypic differences among the groups, we plotted the distribution of the 11 morphological indicators across the five clusters (**Supplementary Figure S2**). The HAR and AR groups exhibited higher values of GP, GR, GI, VI, RL, SL, RFW, SFW, RDW, and SDW, reflecting superior early germination, seedling vigor, and biomass accumulation under alkaline stress. In contrast, the MAS and HAS groups showed lower values in these traits, indicating weaker growth performance and greater sensitivity. Overall, the indicator distribution patterns support the validity of the clustering and demonstrate that these traits can effectively differentiate maize accessions according to their alkali tolerance.

### 3.8 Discriminant analysis for maize material classification

Fisher’s discriminant analysis based on Bayesian coefficients was used to validate the accuracy of the clustering results and the alkali tolerance groupings (**Figure 5B**). Under alkaline salt stress, the HAR group exhibited the highest projected scores, followed by the AR and MAR groups, which displayed intermediate scores, while the MAS and HAS groups showed the lowest scores. Comparison of the discriminant function with the squared Euclidean distance method revealed a 92.9% concordance, indicating high reliability. The overall accuracy of the classification reached 95.2%, with a misjudgment rate of 4.8%, further demonstrating the robustness and precision of the evaluation approach.

### 3.9 Morphological variation analysis among the alkali-tolerant, alkali-sensitive, and overall maize materials

**Figure 6:**
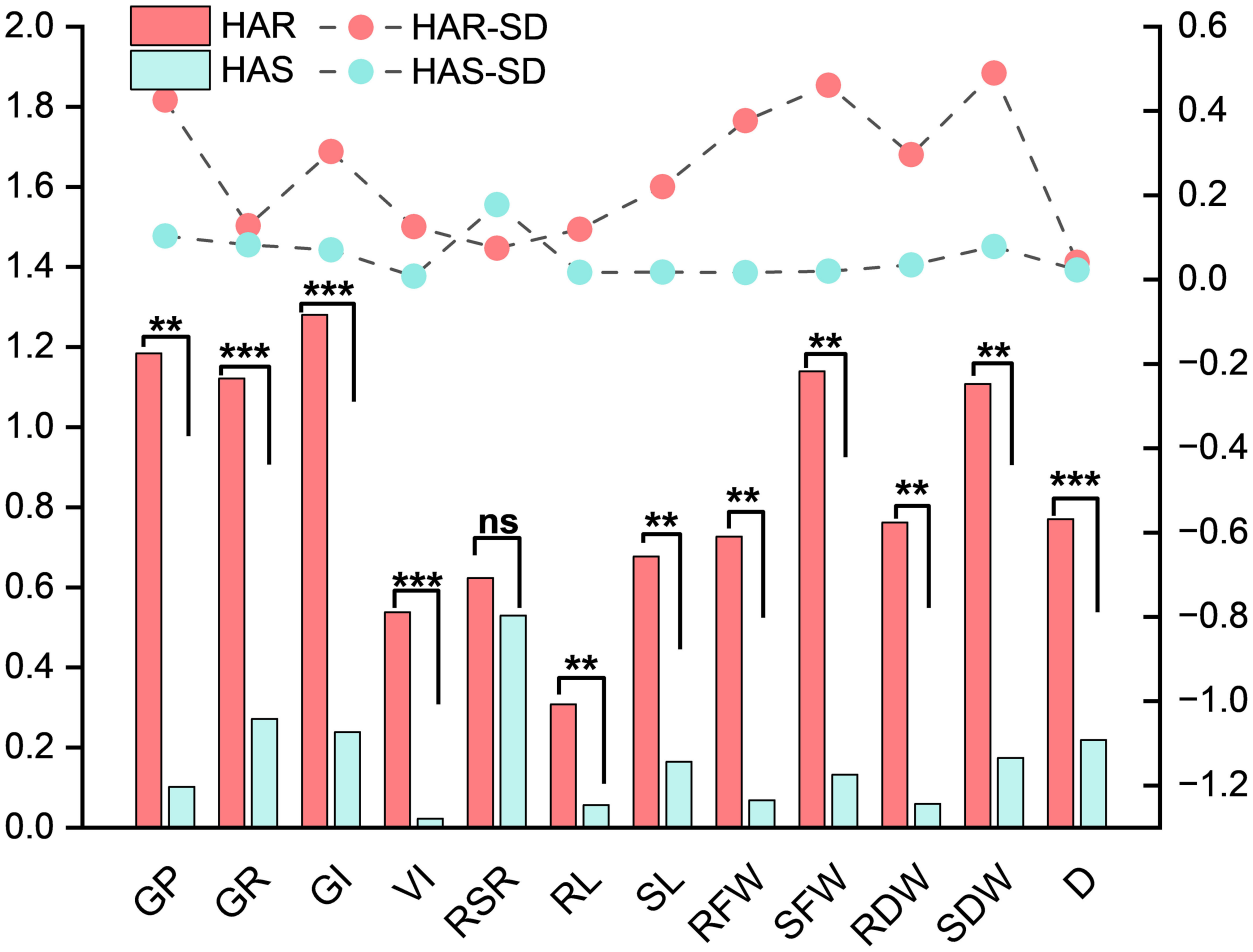
Comparisons of the alkali tolerance coefficients (ATCs) for the germination potential, germination rate, germination index, vigor index, root-to-shoot ratio, root length, shoot length, root fresh weight, shoot fresh weight, root dry weight, shoot dry weight, and *D* value under stress in five highly alkali-tolerant and five highly alkali-sensitive maize materials using mean values. *p < 0.05, **p < 0.01, ***p < 0.001. ns, not significant.

A paired-samples *t*-test was conducted using the mean values under the control and alkaline conditions for the HAR and HAS groups and the full set of accessions (**Table 8**). In the HAR group, majority of the traits were not significantly affected by alkaline stress. GP, GR, and GI showed slight increases under stress, reaching values of 0.280, 0.660, and 19.73, respectively, with all *p*-values greater than 0.05. Similarly, SL, RFW, SFW, RDW, and SDW exhibited minor changes without statistical significance. Only RL and RSR showed notable reductions, decreasing to 1.467 cm and 0.869, respectively (*p* < 0.05), indicating that the root elongation traits were primarily affected in tolerant lines. In contrast, the HAS group displayed pronounced and highly significant declines across all morphological indicators (*p* < 0.01). GP, GR, and GI dropped to 0.040, 0.187, and 4.36, respectively, while VI decreased sharply to 3.44. RL and SL were reduced to 0.290 cm and 0.492, respectively, and both the root and shoot fresh and dry biomass declined by over 80%–90%, demonstrating the severe susceptibility of the HAS lines to high-pH stress. Analysis of the full dataset revealed similar trends, with all 11 traits showing highly significant reductions under alkaline conditions (*p* < 0.01). **Table 8** provides the mean values and the *t*-test results for clear numerical comparisons. The observed differences in the tolerance coefficients between the HAR and HAS groups (**Figure 6**) further highlighted the contrasting phenotypic responses, supporting the identification of alkali-tolerant accessions. .

**Table 8:** Comparison of the various parameters between the alkali-treated and untreated groups using paired-samples ***t***-tests.

Variable		HAR				HAS				Total			
		*N*	Mean (SD)	*t*	*p*	*N*	Mean (SD)	*t*	*p*	*N*	Mean (SD)	t	p
GP	No alkali	5	0.253 (0.096)	−0.55	0.61	5	0.407 (0.132)	6.15	0.00**	42	0.415 (0.196)	8.031	0.00**
	Alkali	5	0.28 (0.084)			5	0.04 (0.043)			42	0.161 (0.142)		
GR	No alkali	5	0.593 (0.128)	−2.39	0.08	5	0.707 (0.126)	7.94	0.00**	42	0.753 (0.159)	9.962	0.00**
	Alkali	5	0.66 (0.13)			5	0.187 (0.051)			42	0.415 (0.217)		
GI	No alkali	5	16.127 (4.236)	−2.83	0.05	5	19.187 (4.323)	6.72	0.00**	42	19.937 (5.505)	7.79	0.00**
	Alkali	5	19.733 (2.946)			5	4.36 (0.84)			42	11.236 (6.6)		
VI	No alkali	5	117.009 (77.846)	2.43	0.07	5	163.254 (51.694)	6.94	0.00**	42	127.081 (72.974)	9.605	0.00**
	Alkali	5	58.175 (25.98)			5	3.442 (0.896)			42	22.015 (20.358)		
RSR	No alkali	5	1.383 (0.231)	10.95	0.00**	5	1.129 (0.177)	5.28	0.01*	42	1.273 (0.246)	6.882	0.00**
	Alkali	5	0.869 (0.217)			5	0.588 (0.162)			42	0.849 (0.304)		
RL	No alkali	5	4.881 (2.636)	3.92	0.02*	5	5.425 (1.442)	8.01	0.00**	42	4.16 (1.841)	12.019	0.00**
	Alkali	5	1.467 (0.908)			5	0.29 (0.039)			42	0.772 (0.514)		
SL	No alkali	5	2.39 (1.048)	1.97	0.12	5	3.048 (0.801)	7.89	0.00**	42	2.131 (0.824)	7.772	0.00**
	Alkali	5	1.475 (0.428)			5	0.492 (0.08)			42	1.001 (0.4)		
RFW	No alkali	5	0.089 (0.028)	1.32	0.26	5	0.11 (0.039)	6.08	0.00**	42	0.088 (0.039)	7.95	0.00**
	Alkali	5	0.069 (0.047)			5	0.007 (0.002)			42	0.034 (0.027)		
SFW	No alkali	5	0.064 (0.015)	−0.67	0.54	5	0.097 (0.028)	7.73	0.00**	42	0.068 (0.027)	5.69	0.00**
	Alkali	5	0.073 (0.032)			5	0.013 (0.004)			42	0.037 (0.023)		
RDW	No alkali	5	0.009 (0.004)	1.25	0.28	5	0.012 (0.004)	6.38	0.00**	42	0.009 (0.004)	6.42	0.00**
	Alkali	5	0.008 (0.006)			5	0.001 (0)			42	0.004 (0.003)		
SDW	No alkali	5	0.007 (0.001)	0.858	0.44	5	0.008 (0.001)	9.853	0.00**	42	0.007 (0.003)	6.054	0.00**
	Alkali	5	0.006 (0.001)			5	0.001 (0.001)			42	0.004 (0.002)		

*GP*, germination potential; *GR*, germination rate; *GI*, germination index; *VI*, vigor index; *RSR*, root-shoot ratio; *RL*, root length; *SL*, shoot length; *RFW*, root fresh weight; *SFW*, shoot fresh weight; *RDW*, root dry weight; *SDW*, shoot dry weight; *HAR*, highly alkali-resistant; *HAS*, highly alkali-sensitive.

*p < 0.05, **p < 0.01, ***p < 0.001.

## 4 Discussion

### 4.1 Contribution of the morphological traits under alkaline stress

To identify the key morphological traits that contribute to maize alkalinity tolerance, a correlation analysis was first performed, selecting the traits significantly associated with the *D* value (i.e., GP, GR, GI, VI, RL, SL, RFW, SFW, RDW, and SDW) while excluding the unrelated trait, i.e., RSR. Stepwise regression was then conducted using these 10 traits as the independent variables and the *D* value as the dependent variable to determine the most influential indicators. Subsequently, the LMG method was applied to quantify the relative contributions of these key traits. The results revealed GI, SDW, RL, and RFW as the major contributors, jointly explaining over 40% of the total variance. GI and SDW reflect early seedling vigor, while RL and RFW indicate root system development and resource acquisition capacity. Notably, the root-related traits alone contributed approximately 26% of the total *R*^2^, highlighting their essential role in sustaining growth under high-pH conditions. These findings are consistent with previous studies reporting that an enhanced root elongation and biomass improve saline–alkali tolerance in maize and other crops (Khan et al., 2024b).

To complement LMG, the standardized regression coefficients (*β*) were analyzed. While *β* values quantify direct effects, they can be influenced by multicollinearity, whereas LMG decomposition averages the contribution of each variable across all possible model orderings, providing an unbiased estimate. Both approaches yielded consistent results, with GI and SDW showing the highest values, followed by RL and RFW, confirming the dominant roles of seedling vigor and root performance in the maintenance of growth under alkaline stress.

### 4.2 Morphological changes under alkaline stress

Building on the identification of the key traits associated with alkalinity tolerance, it is necessary to conduct a comprehensive analysis of the morphological adaptations of maize under alkaline stress. Maize is highly sensitive to salinity and alkalinity, and excessive soil alkalinity can impede water uptake, thereby affecting growth and biomass accumulation. Natural maize populations exhibit considerable genetic diversity, providing a basis for the screening of tolerant lines (Zhang et al., 2019; Zhao et al., 2020; Luo et al., 2021). In this study, alkaline stress markedly affected the germination and early seedling traits, with coefficients of variation ranging from 45% to 95%. The tolerant lines showed minimal reductions, limited to RL and RSR, whereas the sensitive lines exhibited significant decreases across nearly all traits (*t*-test: *p* < 0.001). These observations are highly consistent with the contribution rates of the key traits identified by the LMG analysis, further confirming the importance of SDW and RL in the evaluation of alkalinity tolerance and providing a reliable basis for subsequent *D*-value-based comprehensive assessment.

The root-related traits, particularly RL and RFW, are critical indicators for the screening of stress-tolerant maize (Yu et al., 2021; Sun et al., 2022; Ru et al., 2024), and morphological changes under stress have been extensively characterized (Yu et al., 2021). An elevated soil pH adversely affects root development, reducing the root vitality and limiting the water and nutrient uptake, thereby impairing plant growth (Bibikova et al., 1998; Chen et al., 2021). As the primary organ that perceives stress signals, the maize root system including the primary and lateral roots plays a central role in adaptation. Salt and alkaline stresses inhibit root elongation and biomass accumulation, whereas an enhanced tolerance may involve promoting lateral root proliferation while moderating primary root elongation (Rewald et al., 2012; Bellini et al., 2014; Koevoets et al., 2016). Changes in the RL, biomass, and branching patterns form a crucial physiological basis for the adaptation of maize to alkaline stress (Julkowska et al., 2014; Li et al., 2021).

Furthermore, alkaline soils often cause more severe root damage than saline soils (Pan et al., 2020), and species such as bitter beans, sea buckthorn, and black wheatgrass have demonstrated that root traits are key determinants of tolerance (Matsuoka et al., 2022; Wang et al., 2022). Molecular studies have shown that maize can enhance tolerance by regulating gene expression to control the Na^+^ efflux from the roots (Cao et al., 2020). Collectively, these findings highlight that root morphological plasticity is a central adaptive mechanism underpinning the resilience of maize under alkaline stress. Building on this understanding of the root and shoot traits under alkaline conditions, we further examined how these morphological variations reflect the underlying physiological and biochemical disruptions during early seedling development. Alkaline stress imposes multiple simultaneous constraints on early seedling growth. High-pH soil conditions may inhibit water imbibition by reducing cell wall acidification and extensibility, impair the root plasma membrane function, and inhibit the enzyme activities necessary for seed germination and growth (Chen et al., 2017; Yang et al., 2024). Collectively, these processes delay radicle emergence and weaken seedling establishment. Moreover, excessive bicarbonate and carbonate ions interfere with nutrient availability and ion uptake, particularly restricting Ca^2+^, Mg^2+^, and Fe^2+^ acquisition, which in turn limits the root elongation and biomass accumulation (Wang et al., 2022; Kumar et al., 2024). The patterns observed in this study reduced the RL, diminished the fresh and dry weights, and inhibited the shoot growth in the sensitive accessions, which align with these physiological disruptions, indicating that the early morphological traits directly reflect the biochemical and cellular consequences of alkaline stress. The strong correspondence between the morphological variations and the physiological expectations reinforces the biological relevance of the 11 measured traits, explaining why RL, RFW, and SDW exhibited high correlations with the comprehensive *D* value and contributed substantially to the tolerance classification (Lin et al., 2019; Ergun et al., 2024; Guo et al., 2024).

These physiological responses to alkalinity are not unique to maize: they have been widely documented across other species, supporting the generality of the mechanisms observed here. For example, wheat display significant reductions in root biomass and an impaired lateral root development under high-pH stress (Lin et al., 2019), mirroring the patterns observed in maize. Oilseed rape and alfalfa exhibit similar declines in GR and seedling vigor due to bicarbonate-induced osmotic and ionic disturbances. Studies in halophytes such as quinoa and *Puccinellia tenuiflora* have further demonstrated that tolerance is associated with maintaining root growth, sustaining water absorption (Liu et al., 2018; Ye et al., 2019; Zhang et al., 2023; Bao et al., 2024; Xu et al., 2025), and moderating the ion imbalance physiological strategies, consistent with the superior root performance of the tolerant maize accessions in this study. Similar mechanisms have also been reported in rice and soybean, where tolerant cultivars maintained root growth, regulated the carbon and nitrogen metabolism, and modulated the ion and osmotic balance under alkaline stress (Zhang et al., 2016, 2017; Zhou et al., 2023; Xie et al., 2024; Zhang et al., 2025). Collectively, these cross-species comparisons highlight the importance of root development and early seedling vigor as universal indicators of alkalinity tolerance.

### 4.3 Comprehensive evaluation of alkali tolerance in 42 maize materials

Comprehensive evaluation of maize alkali tolerance is essential for germplasm screening and breeding program development. The *D*-value method is a widely used approach for the assessment and differentiation of alkali tolerance among maize genotypes (Khan et al., 2024a; Ren et al., 2025). In this study, 11 morphological traits—GP, GR, GI, VI, RSR, RL, SL, RFW, SFW, RDW, and SDW—were selected for the PCA to reduce dimensionality. PCA was combined with MFA, CA, and LDA to establish a comprehensive evaluation framework for maize alkali tolerance. Using this framework, 42 maize materials were classified into five tolerance levels: highly tolerant (5 accessions), tolerant (6 accessions), moderately tolerant (6 accessions), moderately sensitive (18 accessions), and highly sensitive (7 accessions). The correlation analysis indicated significant associations between the *D* value and 10 of the 11 traits, except for the RSR. Based on these results, the stepwise regression identified six key indicators—GI, SDW, RFW, GP, GR, and RL—which were used to construct a predictive model (Equation 12) for the evaluation of alkali tolerance during the germination stage.

Several maize materials in this study exhibited enhanced germination under mild alkaline stress, consistent with previous reports (Xu et al., 2020). This suggests that, when the stress intensity is below a harmful threshold, moderately alkaline conditions may stimulate seed germination. The observed pattern of stimulation at low concentrations and inhibition at high concentrations reflects a concentration-dependent response, likely related to cellular physiological regulation. At low concentrations, alkaline solutions may enhance the metabolic activity and water uptake, thereby accelerating germination (Guo et al., 2008). In contrast, high concentrations primarily inhibit germination through osmotic stress and ion toxicity (Zhang et al., 2024). These results indicate that the effects of alkaline stress on early maize growth are not unidirectional, but represent a dynamic balance between stimulation and inhibition, consistent with findings in other plant species (Guo et al., 2008).

Seed germination and seedling establishment require an appropriate pH, as both excessively high and low pH values are detrimental to growth (Jefferson et al., 1989; Rivard and Woodard, 1989). A low pH can inhibit germination and early seedling development (Shelley et al., 2018), whereas a high pH disrupts cellular homeostasis, ion balance, and enzyme activity; damages the cellular structures; and interferes with metabolism, ultimately leading to tissue degradation or seed death (Guo et al., 2010). Therefore, maintaining an optimal pH is crucial for maize growth under saline–alkali stress and provides insights into the physiological mechanisms underlying alkali tolerance, guiding future breeding efforts.

Notably, Xianyu335 was classified as alkali-sensitive in this study, in agreement with earlier observations (Zhang, 2016). Nevertheless, contrasting reports have described this variety as tolerant to salt–alkali during the germination and seedling stages (Liu et al., 2023), indicating possible differences in the stress responses across developmental stages or testing environments. This discrepancy may reflect differences in the developmental stage responses (Morton et al., 2019) or the specific stress regime applied here (Na_2_CO_3_/NaHCO_3_ = 1:9, pH 9.21 ± 0.1), which may have exerted stronger effects. These observations further support that plants often experience more severe damage from alkaline stress than from salt stress (Li and Yang, 2023; Qi et al., 2024).

The analytical framework used in this study integrated complementary statistical approaches to capture different aspects of the data structure. PCA reduces the trait dimensionality and extracts principal variation axes, while MFA balances the trait groups to avoid bias from the traits with larger variance. The *D* value synthesizes the PCA–MFA outputs into a single tolerance index. CA subsequently identifies natural grouping patterns among accessions, while LDA validates the classification and improves the discrimination accuracy. The stepwise regression and LMG analyses further quantified the relative contributions of the individual traits. These interconnected methods formed a coherent evaluation system in which dimensionality reduction, classification, and trait attribution mutually support the robustness of the final tolerance assessment. This integrated design strengthens the biological interpretability and reproducibility of the classification outcomes.

### 4.4 Limitations and future perspectives

Although this study established a comprehensive morphological framework for maize alkali tolerance at the germination stage, only the early-stage traits were assessed and physiological or ionomic measurements were not included, limiting mechanistic insights. The evaluation was conducted under controlled laboratory conditions with a single alkaline concentration, which may not fully capture field variability. Future studies should integrate multi-omics approaches and high-throughput phenotyping, as well as validate candidate tolerant accessions under diverse field conditions. Combining morphological, physiological, and molecular data will enhance the robustness and applicability of the tolerance evaluation system for breeding programs.

## 5 Conclusion

This study established a systematic framework for the evaluation of alkali tolerance in maize during germination. Using this framework, eight highly tolerant and seven highly sensitive accessions were identified. By quantifying the contribution of key indicators through the LMG method, GI, SDW, RL, and RFW were confirmed as reliable morphological traits for distinguishing tolerant and sensitive accessions. The results demonstrate that the proposed evaluation system effectively achieves the primary objectives of the study: to identify alkali-tolerant genotypes and determine the key traits associated with tolerance. Overall, this framework provides a practical and robust tool for the rapid screening of maize germplasm and lays a solid foundation for subsequent physiological and breeding studies.

## Data Availability

The original contributions presented in the study are included in the article/supplementary material. Further inquiries can be directed to the corresponding author.
