# Supplementary material for: Comprehensive evaluation of maize germplasm for alkali tolerance during germination
Source: Front Plant Sci. 2026 Jan 12;16:1728607. doi: 10.3389/fpls.2025.1728607 (PMC12832809; doi:10.3389/fpls.2025.1728607)
Supplement: Supplementary file 1 [file DataSheet1.pdf]

Supplementary Material

1 Supplementary Figures

The following are the figures mentioned in the article.

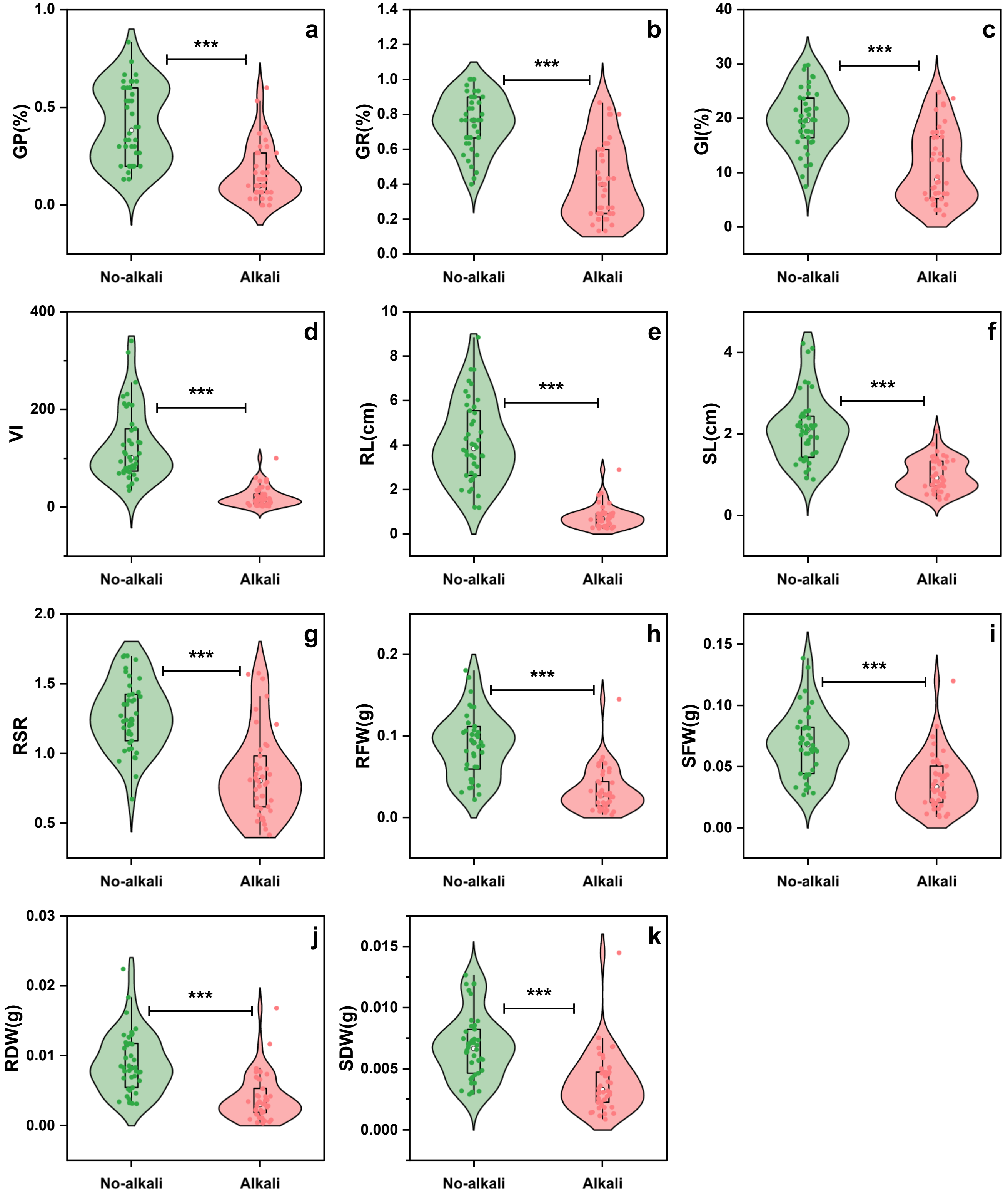

Figure 1. The germination potential(a), germination rate(b), germination index(c), vigor index(d), root-shoot ratio(e), root length(f), shoot length(g), root fresh weight(h), shoot fresh weight(i), root dry weight(j), shoot dry weight(k) of 42 maize samples were determined under alkaline stress and in

a control group. *Significant at the P < 0.05, **Significant at the P < 0.01, ***Significant at the P < 0.001.

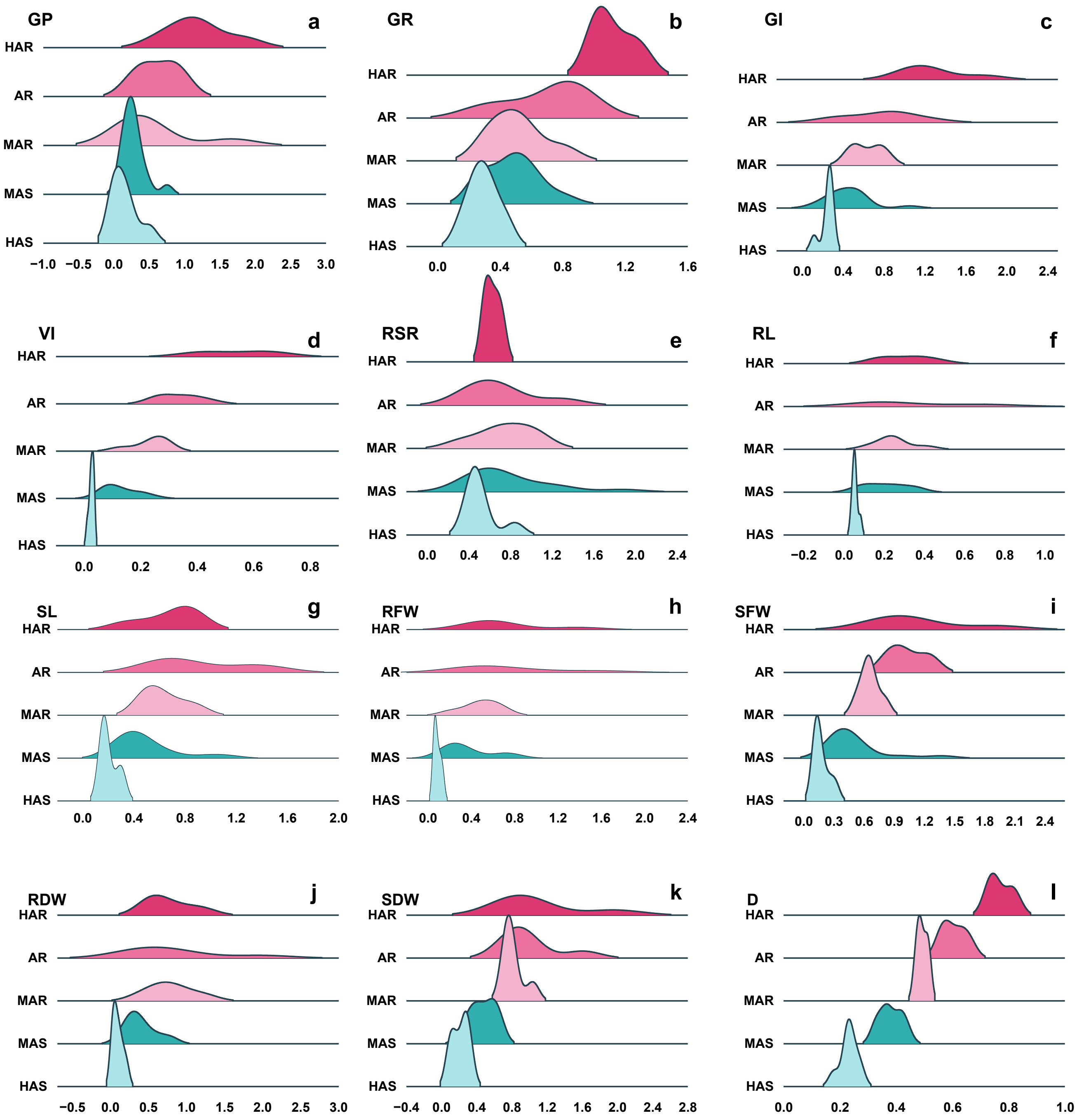

Figure 2. Distribution of alkalinity tolerance coefficients across maize germplasm resources stratified by tolerance group. The germination potential(a), germination rate(b), germination index(c), vigor index(d), root-shoot ratio(e), root length(f), shoot length(g), root fresh weight(h), shoot fresh weight(i), root dry weight(j), shoot dry weight(k) of 42 maize samples.
